# Effects of Moxa (*Folium Artemisiae argyi*) Smoke Exposure on Heart Rate and Heart Rate Variability in Healthy Young Adults: A Randomized, Controlled Human Study

**DOI:** 10.1155/2013/510318

**Published:** 2013-05-23

**Authors:** Yingxue Cui, Baixiao Zhao, Yuhai Huang, Zhanghuang Chen, Ping Liu, Jian Huang, Lixing Lao

**Affiliations:** ^1^School of Acupuncture and Moxibustion and Tuina, Beijing University of Chinese Medicine, Beijing 100029, China; ^2^Chinese Astronaut Scientific Research and Training Center, Beijing 100094, China; ^3^Department of Family and Community Medicine, Center for Integrative Medicine, School of Medicine, University of Maryland, Baltimore, MD 21201, USA

## Abstract

*Objective*. To determine the effects of the moxa smoke on human heart rate (HR) and heart rate variability (HRV). *Methods*. Fifty-five healthy young adults were randomly divided into experimental (*n* = 28) and control (*n* = 27) groups. Experimental subjects were exposed to moxa smoke (2.5 ± 0.5 mg/m^3^) twice for 25 minutes in one week. ECG monitoring was performed before, during, and after exposure. Control subjects were exposed to normal indoor air in a similar environment and similarly monitored. Followup was performed the following week. Short-term (5 min) HRV parameters were analyzed with HRV analysis software. SPSS software was used for statistical analysis. *Results*. During and after the first exposure, comparison of percentage changes or changes in all parameters between groups showed no significant differences. During the second exposure, percentage decrease in HR, percentage increases in lnTP, lnHF, lnLF, and RMSSD, and increase in PNN50 were significantly greater in the experimental group than in control. *Conclusion*. No significant adverse HRV effects were associated with this clinically routine 25-minute exposure to moxa smoke, and the data suggests that short-term exposure to moxa smoke might have positive regulating effects on human autonomic function. Further studies are warranted to confirm these findings.

## 1. Introduction

Moxibustion, one of the classical therapies of Traditional Chinese Medicine (TCM), uses the heat generated by burning moxa floss (usually made by *Folium Artemisiae argyi*) to stimulate acupuncture points [1]. It is used widely in acupuncture clinics in China and other Asian countries to treat various diseases, especially chronic conditions such as osteoarthritis, asthma, gastrointestinal disorders, and insomnia [2–5]. Smoke is an unavoidable aspect of the therapy. The aim of this study was to evaluate the effects of moxa smoke exposure on human heart rate variability (HRV) parameters. 

A moxibustion session typically lasts 20–30 minutes, and patients are often treated several times a week for several weeks. Patients are exposed to the smoke during treatment, while acupuncturists are commonly exposed for prolonged periods during clinical practice. Because of recent concerns as to the safety of the therapy, specifically the potential toxicity of the smoke, many clinics no longer use moxibustion, thus depriving patients of the benefits of this unique treatment. Evaluation of the safety and the effects of moxa smoke is imperative.

Concerns about moxa smoke are similar to those regarding tobacco smoke and air pollutants. Many studies show that exposure to tobacco smoke and air pollutants is positively associated with adverse effects in the respiratory, immune, nervous, and cardiovascular systems [6–11]. Active and passive exposures to tobacco smoke have been found to increase sympathetic nervous system activity and reduce parasympathetic nervous system modulation and HRV [12–14]. Particulate air pollutants affect heart rate (HR), blood pressure, vascular tone, blood coagulation, the progression of atherosclerosis [15], and HRV [16–19]. To our knowledge, the influence of moxa smoke on HRV in the human body has not been sufficiently investigated. 

HRV refers to the time variation coefficient between successive heart beat cycles. It is one of the most promising quantitative markers of autonomic nerve system activity [20]. There is a growing recognition of the role of HRV abnormalities in cardiovascular disease [21–23], and HRV decrease is a strong predictor of mortality [24]. HRV measurement in time and frequency domains is a convenient, noninvasive tool for autonomic nervous physiology evaluation, and short-term (5-minute) recording gives reliable measurements [25]. The purpose of this study was to determine whether exposure to moxa smoke influences HR and HRV in healthy subjects.

## 2. Methods

### 2.1. Subjects

Participants, most of them students of Beijing University of Chinese Medicine or other nearby universities plus some residents of the area around the University, were recruited between March 2012 and July 2012. The study protocol was approved by the Human Medical Ethics Committee of Beijing University of Chinese Medicine and was registered in the Chinese Clinical Trial Registry (ChiCTR-TRC-12002445). Written informed consent was secured from all participants. 

Inclusion criteria required that subjects be normal and healthy according to the American Society of Anesthesiologists Physical Status Classification System, that is, that they have no organic, physiologic, biochemical, or psychiatric disorders, smoke <5 cigarettes per day [26], and are between the ages of 18 and 50. 

Individuals were excluded if they (1) had a history of addiction to alcohol or drugs, (2) had had contact with moxa smoke within one month of the test, (3) had used medications within two weeks of the test, (4) had had a cold or other illnesses within one week of the test, (5) had ingested food or drink containing caffeine or alcohol, smoked, or done strenuous exercise within four hours of the test, and (6) were pregnant or lactating.

Participants were instructed to refrain from tobacco, alcohol, medications, and strenuous exercise and to avoid contacting moxa smoke or any other abnormal gas during the two-week test.

### 2.2. Study Protocol

#### 2.2.1. Equipment and Setting

The trial was performed at the Beijing University of Chinese Medicine in two adjacent, bright, quiet, and similarly laid-out rooms equipped with beds. Ambient temperature and humidity were kept between 24°C*∼*26°C and 40%*∼*50% and monitored by a meteorological parameter recorder (Kestrel NK3000, USA). 

Room 1 had normal indoor air. In Room 2, moxa smoke was generated by burning moxa sticks (three-year-old pure moxa, 1.8 cm × 20 cm, Nanyang Hanyi Moxa Co., Ltd., China). A digital dust indicator (P5L2C, Binta Green Technology Co., Ltd., Beijing, China) that detects particulate matter <10 *μ*m in diameter (PM_10_) levels and a volatile organic compound (VOC) detector (model no. PGM-7320, kit MiniRAE3000, Rae Systems, Inc., USA) were set beside the participant to monitor the air. In the moxa room, PM_10_ and total VOC levels were kept between 2.5 ± 0.5 mg/m^3^ and 4.2 ± 1.3 mg/m^3^, respectively, which accord with average moxa smoke levels in acupuncture clinics [27]. In Room 1, PM_10_ and total VOC levels detected in this trial were lower than 0.01 and 0.2 mg/m^3^, respectively.

This was a two-arm, open, and randomized study (*N* = 55). After reading and signing the consent form, the participant was assigned to the experiment or control group by computer-generated random allocation. Group assignments were performed by a statistician blinded to the study design. Each assignment was sealed in an opaque envelope that was opened for the respective participant by the investigator prior to treatment.

#### 2.2.2. Experimental Group

**Figure 1:**
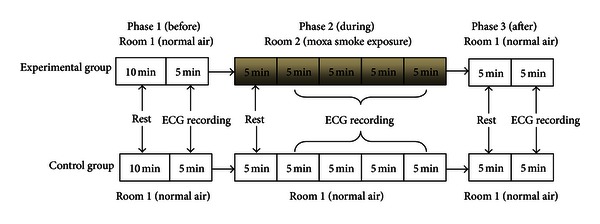
Experimental procedures for tests 1 and 2.

Testing consisted of three phases, one immediately after the other. In phase 1, subjects entered Room 1 and were encouraged to relax in a supine position. After 5–10 minutes of rest, ECG monitoring was performed for 5 minutes. In phase 2, they entered Room 2. After a 5-minute rest in a supine position, ECG monitoring was performed for 20 minutes. In phase 3, they returned to Room 1 for another 5-minute ECG recording (see Figure 1). After each ECG, subjects recorded their subjective sensations and emotions on a questionnaire. These questions were about whether they have experienced drowsiness, shortness of breath, cough, choking, irritation in nose, pharynx, and eyes, body temperature changes, or any other discomfort that might be associated with moxa smoking exposure.

#### 2.2.3. Control Group

Control subjects were similarly monitored but remained in Room 1 during phase 2 (see Figure 1). 

**Figure 2:**
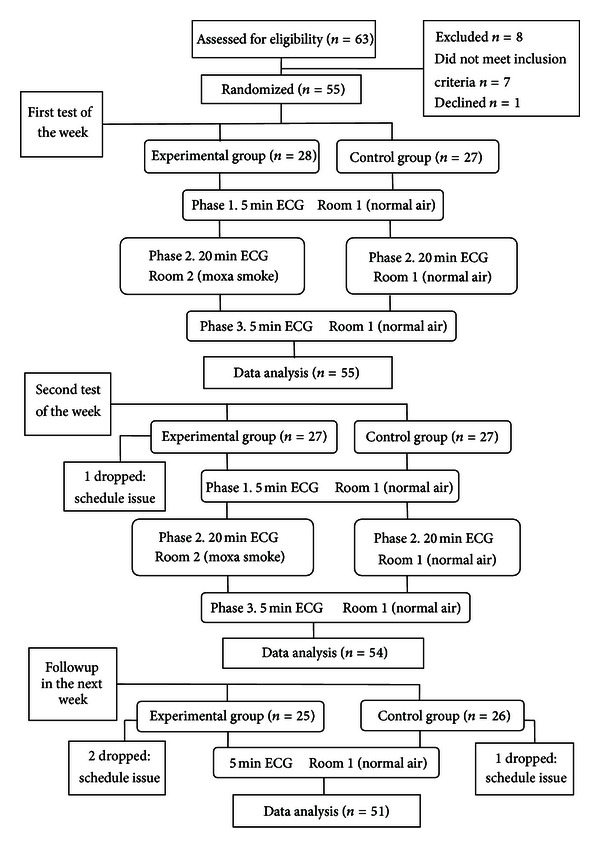
Flow of participants through the whole trial.

The test was performed on each subject twice in a single week to accord with routine clinic practice. One week later, subjects in both groups returned for another 5-minute ECG (see Figure 2).

### 2.3. ECG Monitoring and Short-Term (5 min) HRV Data Analysis

With the subjects supine, three ECG electrodes were placed on their right subclavian and double costal arch regions. A data acquisition instrument (DATAQ Instrument Inc., MODEL:DI-720-USB, USA) was connected to the electrodes and a computer. To allow the heart beat to become steady, ECG recording was started 5–10 minutes after they lay down.

ECGs were analyzed by a specialist blinded to group assignment. After removal of extraneous noise, normal-to-normal beat intervals were analyzed for time- and frequency-domain parameters in 5-minute epochs using standard algorithms and HRV analysis software (Catholic University of Leuven). Time-domain analysis estimates the variation of differences between successive RR intervals through statistically developed indices. Frequency-domain analysis estimates respiratory-dependent, high- and low-frequency power through spectral analysis. Widely used HRV parameters [25, 28, 29] were employed in this study (Table 1).

### 2.4. Statistical Analysis

SPSS17.0 statistical software was used for data analysis. The paired *t*-test was used to compare HR data of different phases in the same group; the independent sample *t*-test was used to compare data from different groups. A related sample nonparametric test (Wilcoxon) was used to compare all HRV parameter data of different phases in the same group, and an independent sample nonparametric test (Mann-Whitney *U* Test) was used to compare data from different groups. 

TP, HF, and LF data were transformed into natural logarithms (ln) for better analysis. The four-segment data of phase 2 were calculated into one mean value for comparison with data from the other two phases. Percentage changes ((mean value in phase 2/value in phase 3 − value in phase 1)/value in phase 1 × 100%) or changes (mean value in phase 2/value in phase 3 − value in phase 1) of all data were used for comparisons between the groups.

## 3. Results

### 3.1. Baseline Characteristic of Study Subjects

There were no statistically significant baseline differences between the groups (Table 2).

### 3.2. Comparisons: First Test

**Figure 3:**
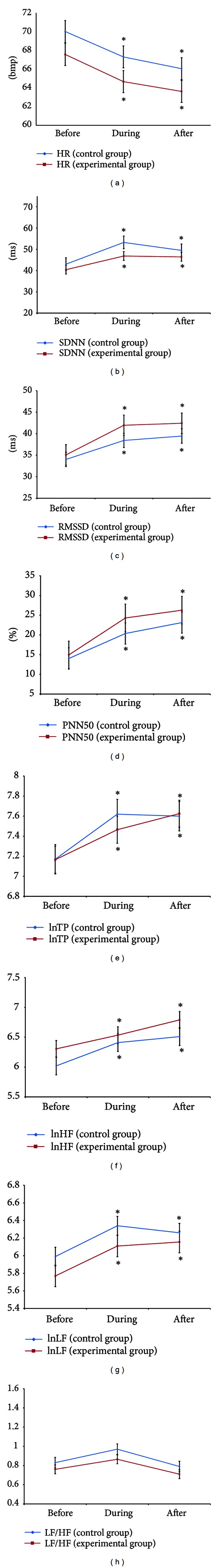
Changes in all indicators in the first test of the week. Values are expressed as (a) mean and (b)–(h) median. * indicates a significant difference (*P* < 0.05) between during/after periods and before periods in the same group using (a) paired *t*-test and (b)–(h) Wilcoxon test.

In phases 2 and 3, during and after exposure, HR (*P* < 0.05) was significantly reduced, and SDNN (*P* < 0.05), RMSSD (*P* < 0.05), PNN50 (*P* < 0.05), lnTP (*P* < 0.05), lnLF (*P* < 0.05), and lnHF (*P* < 0.05) were significantly increased in both groups (Figures 3(a)–3(g)). No significant change was found in LF/HF in either group (Figure 3(h)).

Comparison of the percentage changes/changes in HR and HRV parameters showed no significant differences between groups (Table 3).

### 3.3. Comparisons: Second Test

**Figure 4:**
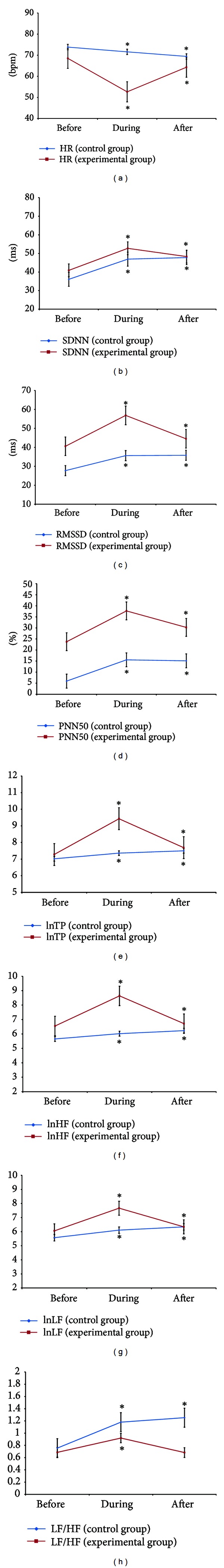
Changes in all indicators in the second test of the week. Values are expressed as (a) mean and (b)–(h) median. *indicates a significant difference (*P* < 0.05) between the during/after phases and phase 1 in each group using (a) paired *t*-test and (b)–(h) Wilcoxon test.

In phases 2 and 3, during and after exposure, each group had significant reductions in HR (*P* < 0.05) and significant increases in all HRV parameters (*P* < 0.05; Figures 4(a)–4(h)) except for LF/HF ratio in phase 3 of the experimental group (Figure 4(h)).

In phase 2, the experimental group's percentage decrease in HR (*P* < 0.001), increases in RMSSD (*P* < 0.001), lnTP (*P* < 0.001), lnHF (*P* < 0.001), and lnLF (*P* < 0.001), and increase in PNN50 (*P* = 0.02) were significantly greater than those in control. In phase 3, LF/HF ratio increase was significantly lower in the experimental group than in control (*P* = 0.005; see Table 4).

### 3.4. Comparisons: Follow-Up Test

Mean HR (*P* = 0.039) in the experimental group was significantly lower thanthatin control; other indicators were not significantly different in the two groups (Table 5).

### 3.5. Participants' Sensations

During moxa smoke exposure, seventeen experimental group subjects felt sleepy and relaxed. One felt refreshed; stomach and bowel movement improved in another. Ten complained of choking and irritation in nose, pharynx, and eyes. One had difficulty in breathing. Eight had no unusual sensations. 

In the control group, two subjects felt sleepy; one had neck discomfort; one had numbness in the hand. Twenty-three felt nothing unusual.

## 4. Discussion 

No harmful HR and HRV effects were observed during exposure to clinical levels of moxa smoke. Evidence for this is that there were no differences in HR and HRV, either immediately (after 10 minutes) or at followup a week after exposure, between the experimental group exposed to moxa smoke and the control group without such exposure. These results might explain why reports of adverse reactions associated with smoke produced in this ancient therapy are so rare. 

In contrast to retrospective studies based on clinical observation, our present study was a well-controlled, randomized, and prospective study to examine possible adverse effects of moxa smoke. The study is unique in moxa smoke concentration, length of exposure to the smoke, and its carefully controlled and monitored experimental environment, all of which mimic actual clinical moxibustion practice. The sample size is comparable to those reported in similar studies on exposure to other types of potentially hazardous smoke [12–14, 30].

The HRV effects that we observed in this moxa smoke study contrast with findings of air pollution and tobacco smoke studies, which show harmful effects on human health [12–19]. This difference might be the result of the unique constituents of moxa smoke. Moxa floss (burning material of moxibustion) is made from the mugwort leaf (*Folium Artemisiae argyi*), and its smoke contains multiple essential oils, suspended particulate matters, and products of chemical oxidation [31]. Wheeler et al. [32] tested the chemical products of moxibustion in clinically common dosages and found that neither carbon monoxide nor volatile compounds that present safety hazards are produced under clinical conditions. Air pollution studies show that the suspended particles in polluted air can reduce HRV by affecting the neurological system and consequently affecting the cardiovascular system by increasing HR and blood coagulation and decreasing hemoglobin to cause oxidative stress [33–35]. The respirable particles in moxa smoke mainly consist of unknown, ultrafine particles [27], which might be one reason why we observed no adverse HRV effects from clinical levels of the smoke. However, further investigation is needed to confirm and refine our finding.

Interestingly, in the second test we observed positive HR and HRV parameter changes in the experimental group compared to control. These include decrease in mean HR and increases in both time-domain analysis HRV (RMSSD, PNN50) and frequency-domain (TP, HF, and LF) during the 25-minute moxa smoke exposure (Table 4). HRV has been widely applied as a marker of autonomic nervous activity. Tension of the autonomic nervous system is maintained by opposing actions of the sympathetic and parasympathetic systems. RMSSD, PNN50, and HF are primarily thought to reflect parasympathetic influences. LF has been shown to reflect both sympathetic and parasympathetic influences. The LF/HF ratio is widely used as a relative marker of sympathetic nervous activities or sympathovagal balance [25, 36]. The HRV changes found in this study appear to be linked to the restorative functions of the autonomic system. These include an increase in total variability shown by increased TP and an increase in parasympathetic nervous activity shown by increased RMSSD, PNN50, and HF. LF/HF increase after moxa smoke exposure was significantly lower in experimental subjects than in control, which may indicate that moxa smoke drives autonomic nervous activity toward a balanced state. These findings are consistent with those of our previous pilot study in which 24 healthy volunteers exposed to moxa smoke had significant reduction in HR and increase in total HRV during and after 20 minutes of exposure to moxa smoke [37]. This suggests that moxa smoke has a regulating effect on human autonomic system function and that moxa smoke inhalation might have short-term stress-alleviating effects. 

Moxa smoke effects and mechanisms have not been well investigated. We speculate that the effects are similar to those of aromatherapy, as a number of studies [38–42] show that inhalation of certain aromas can induce HRV increase and HR reduction, indicating beneficial autonomic nervous system regulation. Mechanisms of these effects might be pharmacological and/or psychological [43]. The pharmacological hypothesis is that the odor directly interacts with and affects the autonomic nervous system/central nervous system and/or endocrine systems. On the one hand, the pharmacological compound might enter the bloodstream by way of nasal or lung mucosa; on the other, the odor might stimulate the olfactory nerves and the limbic system of the brain. In the clinic, moxibustion is often used to treat insomnia, anxiety, and depression [4, 5]. However, it is unclear whether the treatment effects are induced by heat at the acupuncture point or by the moxa smoke. The present study provides some information for distinguishing the respective roles of heat and smoke. Further studies to elucidate the mechanisms of moxibustion are warranted.

We are aware of the limitations of the present study. Our data only show HRV effects from short-term exposure to moxa smoke; in normal acupuncture practice, patients usually receive multiple moxibustion treatments, and practitioners are usually exposed to the smoke for years. These factors warrant a long-term observational study. Furthermore, because the participants in our study were not blinded, we cannot rule out the possibility of placebo effect. Additionally, our subjects were young and healthy; these results might not reflect how moxa smoke affects the elderly or chronically ill.

Nevertheless, this study is an important step toward understanding the effects of moxa smoke. Our results provide useful information on the feasibility of a future, larger trial and will make it possible to calculate adequate sample sizes for such research. 

In conclusion, our data show that short-term moxa smoke exposure at clinical concentrations poses no hazards to patients' HR and HRV and suggest that moxa smoke has a positive regulating effect on human autonomic function. Future studies are needed to further investigate the effects and the safety of moxa smoke.

## Figures and Tables

**Table 1:** HRV parameters used in this trial.

Variable	Units	Description
Time-domain parameters		

SDNN	msec	the standard deviation of all NN intervals, an estimate of overall variability
rMSSD	msec	the square root of the mean of the squared differences between adjacent NN intervals, an estimate of the short-term components of variability
pNN50	%	the proportion derived by dividing NN50 (the number of interval differences of successive normal-to-normal intervals greater than 50 ms) count by the total number of normal-to-normal intervals

Frequency- domain parameters		

TP	msec^2^	total power, frequency range <0.4 Hz
HF	msec^2^	power in the high-frequency range (0.15–0.4 Hz), considered to be mediated mainly by vagal activity
LF	msec^2^	power in the low-frequency range (0.04–0.15 Hz), suggested to be mediated by both sympathetic and parasympathetic activities
LF/HF	Ratio	an indicator of the balance of the sympathetic and parasympathetic systems

**Table 2:** Baseline characteristics of study subjects.

Variables		Experimental group (*N* = 28)	Control group (*N* = 27)
Gender^a^	Male/female	12/16	12/15
Age^ b^	Years	24.5 ± 2.5	25 ± 2.3
BMI^b^	kg/m^2^	21.2 ± 2.9	21.1 ± 2.3
Ethnic group^a^	Han/others	27/1	26/1
Nationality^a^	China/Singapore	26/2	26/1
Smoking history^a^	Yes/no	0/28	0/27
Regular exercise^a^	Yes/no	11/17	7/20
Emotional condition^a^	Good/ok/bad	11/17/0	13/14/0
Mean HR^b^	bpm	67.57 ± 8.95	70.02 ± 7.31
SDNN^c^	ms	40.43 (12.89)	43.02 (26.26)
RMSSD^c^	ms	35.08 (24.80)	34.02 (13.8)
PNN50^c^	%	14.93 (32.12)	14.02 (17.21)
lnTP^c^		7.17 (0.77)	7.17 (1.18)
lnHF^c^		6.31 (1.21)	6.02 (1.36)
lnLF^c^		5.77 (0.8)	5.99 (0.96)
LF/HF^c^		0.76 (0.82)	0.83 (0.72)

^b^Mean ± standard deviation; ^c^median (interquartile range). No significant differences were found between the groups based on ^a^Chi-square test, ^b^independent two-sample *t*-test, or ^c^Mann-Whitney *U*  Test.

**Table 3:** Comparison of the percentage changes/changes in HR and HRV parameters between the groups in the first test of the week.

	Experimental group	Control group	*P* value
HR^1,a^			
Before-during	−0.04 ± 0.03	−0.04 ± 0.04	0.75
Before-after	−0.06 ± 0.05	−0.06 ± 0.04	0.92
SDNN^1,b^			
Before-during	0.19 (0.35)	0.20 (0.29)	0.74
Before-after	0.22 (0.42)	0.14 (0.33)	0.62
RMSSD^1,b^			
Before-during	0.18 (0.38)	0.09 (0.35)	0.78
Before-after	0.18 (0.36)	0.15 (0.42)	0.89
PNN50^2,b^			
Before-during	4.03 (10.34)	4.88 (11.44)	0.56
Before-after	4.32 (8.46)	5.26 (15.3)	0.72
lnTP^1,b^			
Before-during	0.03 (0.09)	0.04 (0.09)	0.58
Before-after	0.05 (0.08)	0.04 (0.07)	0.60
lnHF^1,b^			
Before-during	0.05 (0.1)	0.04 (0.11)	0.46
Before-after	0.05 (0.09)	0.04 (0.11)	0.45
lnLF^1,b^			
Before-during	0.03 (0.15)	0.04 (0.09)	0.57
Before-after	0.03 (0.17)	0.04 (0.18)	0.82
LF/HF^2,b^			
Before-during	0.11 (0.51)	0.09 (0.34)	0.89
Before-after	−0.03 (0.64)	−0.15 (0.85)	0.79

Number 1 indicates percentage changes; number 2 indicates data changes. Values are expressed as ^a^mean ± standard deviation and ^b^median (interquartile range). No significant differences were found between the groups using ^a^independent two-sample *t*-test and ^b^Mann-Whitney *U* test.

**Table 4:** Test 2: percentage changes/changes in HR and HRV parameters.

	Experimental group	Control group	*P* value
HR^1,a^			
Before-during	−0.23 ± 0.02*	−0.03 ± 0.09	<0.001
Before-after	−0.06 ± 0.04	−0.06 ± 0.09	0.63
SDNN^1,b^			
Before-during	0.21 (0.32)	0.15 (0.41)	0.76
Before-after	0.16 (0.36)	0.28 (0.57)	0.56
RMSSD^1,b^			
Before-during	0.42 (0.29)*	0.19 (0.30)	<0.001
Before-after	0.12 (0.37)	0.24 (0.57)	0.47
PNN50^2,b^			
Before-during	9.78 (17.32)^△^	5.02 (7.81)	0.02
Before-after	4.26 (13.59)	10.59 (17.26)	0.34
lnTP^1,b^			
Before-during	0.3 (0.09)*	0.05 (0.08)	<0.001
Before-after	0.04 (0.09)	0.09 (0.13)	0.18
lnHF^1,b^			
Before-during	0.29 (0.07)*	0.03 (0.09)	<0.001
Before-after	0.06 (0.1)	0.06 (0.19)	0.71
lnLF^1,b^			
Before-during	0.31 (0.21)*	0.07 (0.15)	<0.001
Before-after	0.10 (0.19)	0.15 (0.23)	0.15
LF/HF^2,b^			
Before-during	0.18 (0.34)	0.32 (0.84)	0.30
Before-after	0.13 (0.69)^△^	0.44 (1.19)	0.005

Number 1 indicates percentage changes; number 2 indicates data changes. Values are expressed as ^a^mean ± standard deviation and ^b^median (interquartile range). The symbols * and  ^△^ indicate significant differences (*P* < 0.001 and *P* < 0.05, resp.) between the groups using ^a^independent two-sample *t*-Test and ^b^Mann-Whitney *U* Test.

**Table 5:** HR and HRV parameters, follow-up test.

	HR^a^	SDNN^b^	RMSSD^b^	PNN50^b^	lnTP^b^	lnHF^b^	lnLF^b^	LF/HF^b^
Control group	72.92 ± 7.83	44.07 (22.26)	34.01 (21.12)	12.37 (21.9)	7.22 (1.04)	5.94 (1.62)	5.83 (1.04)	0.93 (1.17)
Experimental group	67.68 ± 9.74*	52.72 (26.91)	46.16 (38.15)	29.75 (45.16)	7.56 (1.07)	6.73 (1.54)	6.19 (1.35)	1.01 (1.45)

Values are expressed as ^a^mean ± standard deviation and ^b^median (interquartile range). The symbol *indicates a significant difference (*P < *0.05) between the two groups according to the independent two- sample *t*-test.
